# Supplementary material for: Effects of sequence motifs in the yeast 3′ untranslated region determined from massively parallel assays of random sequences
Source: Genome Biol. 2021 Oct 18;22:293. doi: 10.1186/s13059-021-02509-6 (PMC8522215; doi:10.1186/s13059-021-02509-6)
Supplement: Supplementary file 1 — Additional File 1:. Supplementary Figures and Tables [file 13059_2021_2509_MOESM1_ESM.pdf]

Additional File 1: Supplementary Figures and Tables

For the manuscript**:**

Effects of sequence motifs in the yeast 3′ untranslated region determined from massively-parallel assays of random sequences

Andrew Savinov,[1,4] Benjamin M. Brandsen,[1,2] Brooke E. Angell,[1,5] Josh T. Cuperus[1,*] and Stanley Fields[1,3,*]

[1]Department of Genome Sciences, University of Washington, Box 355065, Seattle, WA 98195

[2]Department of Chemistry and Biochemistry, Creighton University, Omaha, NE 68178

[3]Department of Medicine, University of Washington, Box 357720, Seattle, WA 98195

[4]Present address: Department of Biology, Massachusetts Institute of Technology, Cambridge, MA 02142

[5]Present address: Interdisciplinary Biological Sciences Graduate Program, Northwestern University, Evanston, IL 60208

[*]Correspondence to: Stanley Fields, ph. 206 616-4522, fields@uw.edu,
Josh T. Cuperus, ph. 206 685-3776, cuperusj@uw.edu

Contents:

Figures S1 – S10

Table S1

Supplementary Figures

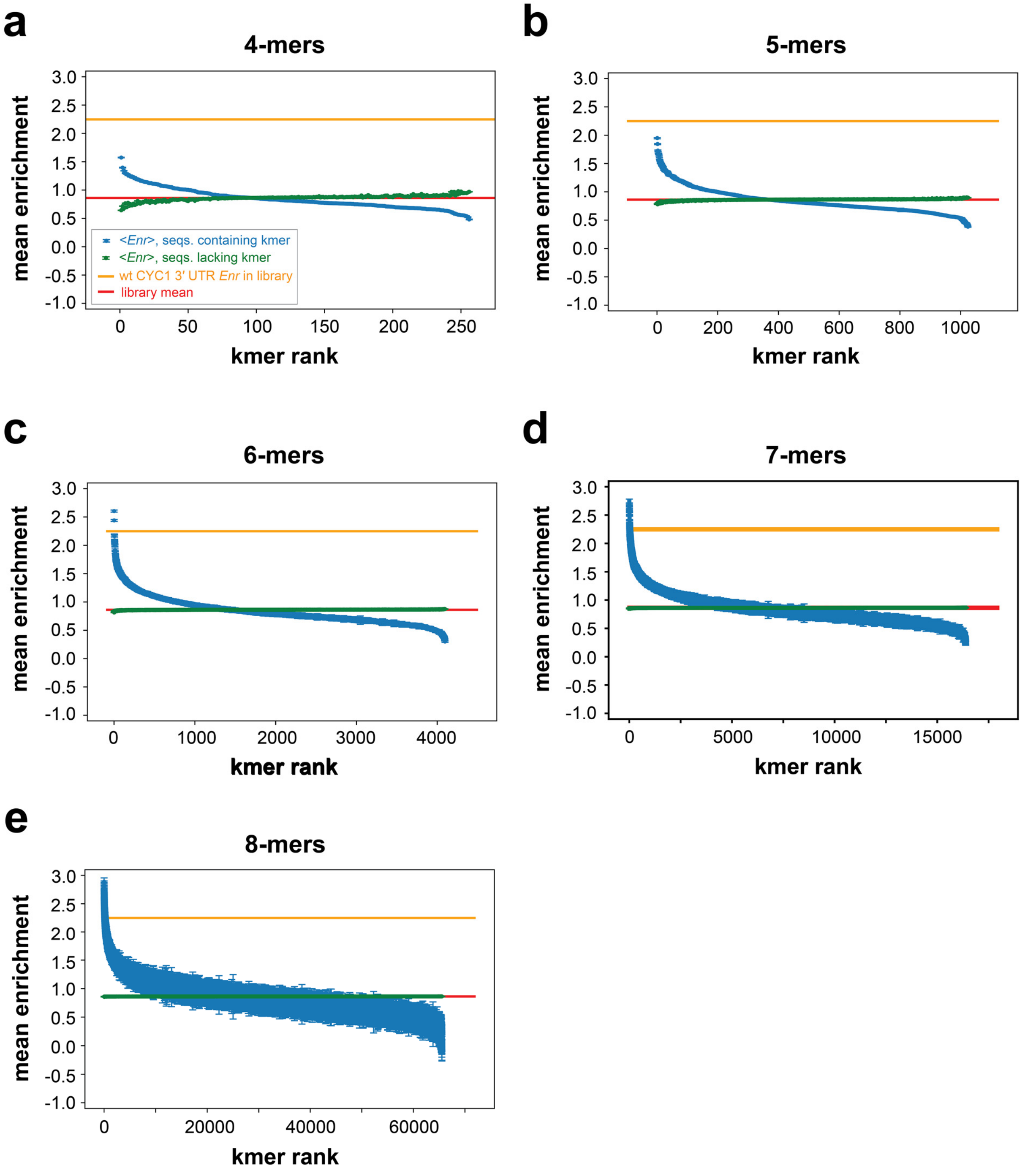

Figure S1. Effects of all possible 4-to-8-mer sequences on protein expression in the N50-C library. (**a**) – (**e**) Average enrichments associated with the indicated *k*-mers and controls as follows. Blue data, average enrichment across all library sequences containing the *k*-mer; green data, average enrichment across all library sequences lacking the *k*-mer; error bars, s.e.m.; red line, average enrichment across all library sequences; orange line, enrichment of plasmid constructs bearing the unaltered *CYC1* 3′ UTR sequence, with no random 50-mer. Error bars indicate s.e.m. The horizontal axis displays *k*-mer sequence "rank" based on level of expression of N50 sequences containing each *k*-mer (i.e., the *k*-mer associated with the highest expression is assigned rank 1).

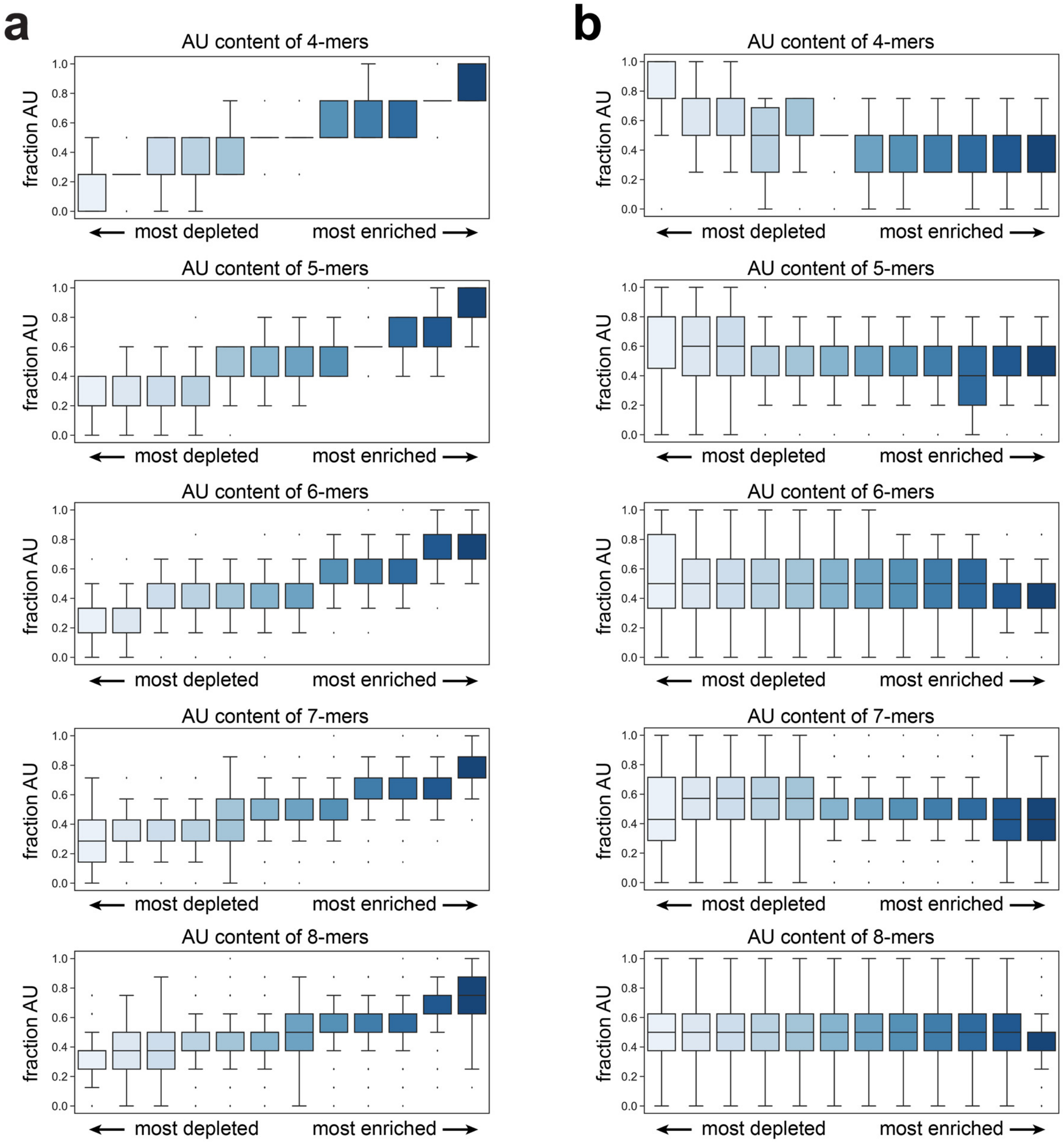

Figure S2. AU content of *k*-mers in the **(a)** N50-C and **(b)** N50-EPC libraries. For each library, *k*-mers of a given length were separated into 12 bins based on the mean enrichment scores of library sequences carrying the given *k*-mer. Bins on the right-hand side of each plot contain *k*-mers found in sequences associated with the highest mean enrichment scores, and bins on the left-hand side of each plot contain *k*-mers associated with the lowest mean enrichment scores.

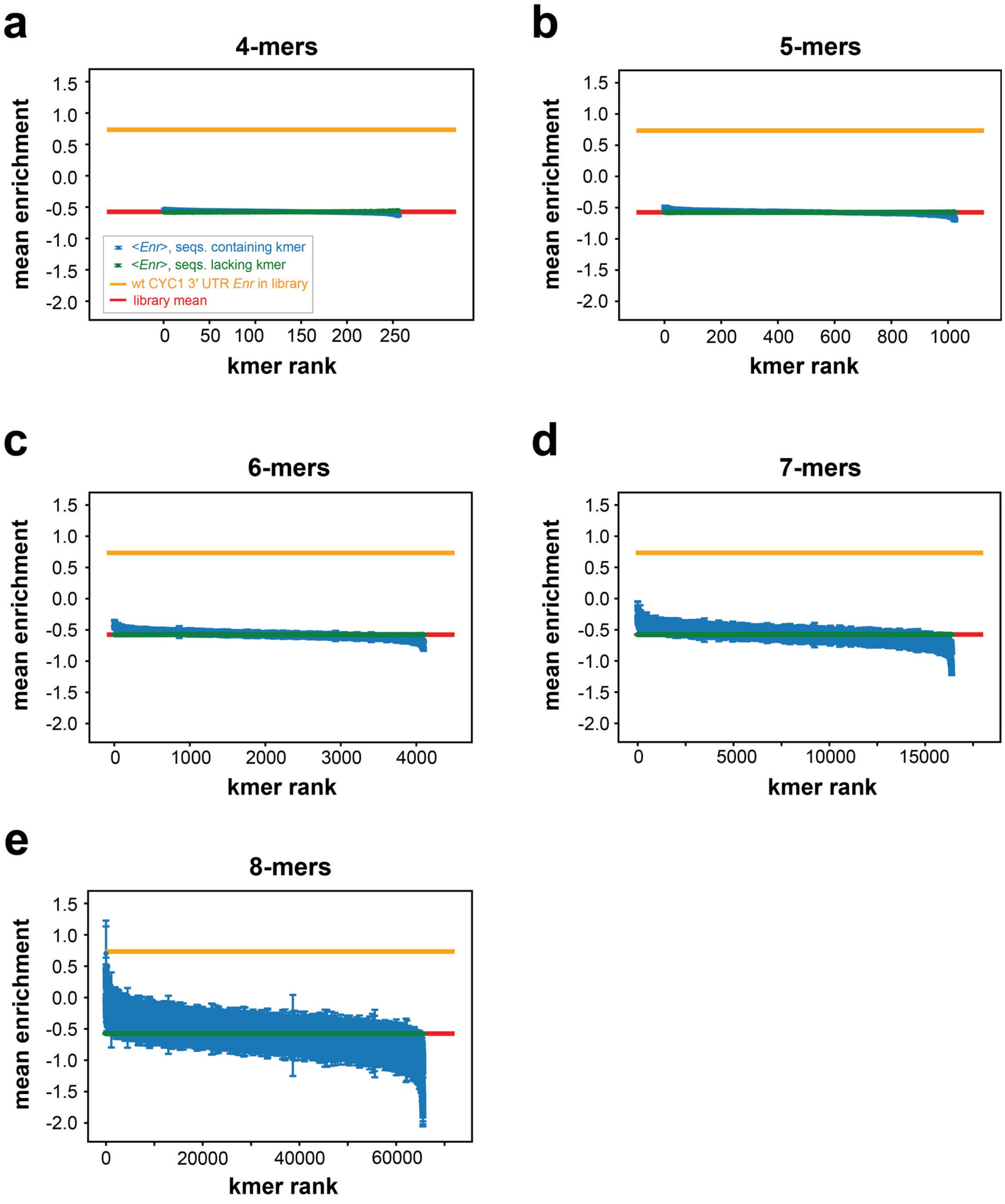

Figure S3. Effects of all possible 4-to-8-mer sequences on protein expression in the N50-EPC library. (**a**) – (**e**) Average enrichments associated with the indicated *k*-mers and controls as follows. Blue data, average enrichment across all library sequences containing the *k*-mer; green data, average enrichment across all library sequences lacking the *k*-mer; error bars, s.e.m.; red line, average enrichment across all library sequences; orange line, enrichment of plasmid constructs bearing the unaltered *CYC1* 3′ UTR sequence, with no random 50-mer. Error bars indicate s.e.m. The horizontal axis displays *k*-mer sequence "rank" based on level of expression of N50 sequences containing each *k*-mer (i.e., the *k*-mer associated with the highest expression is assigned rank 1).

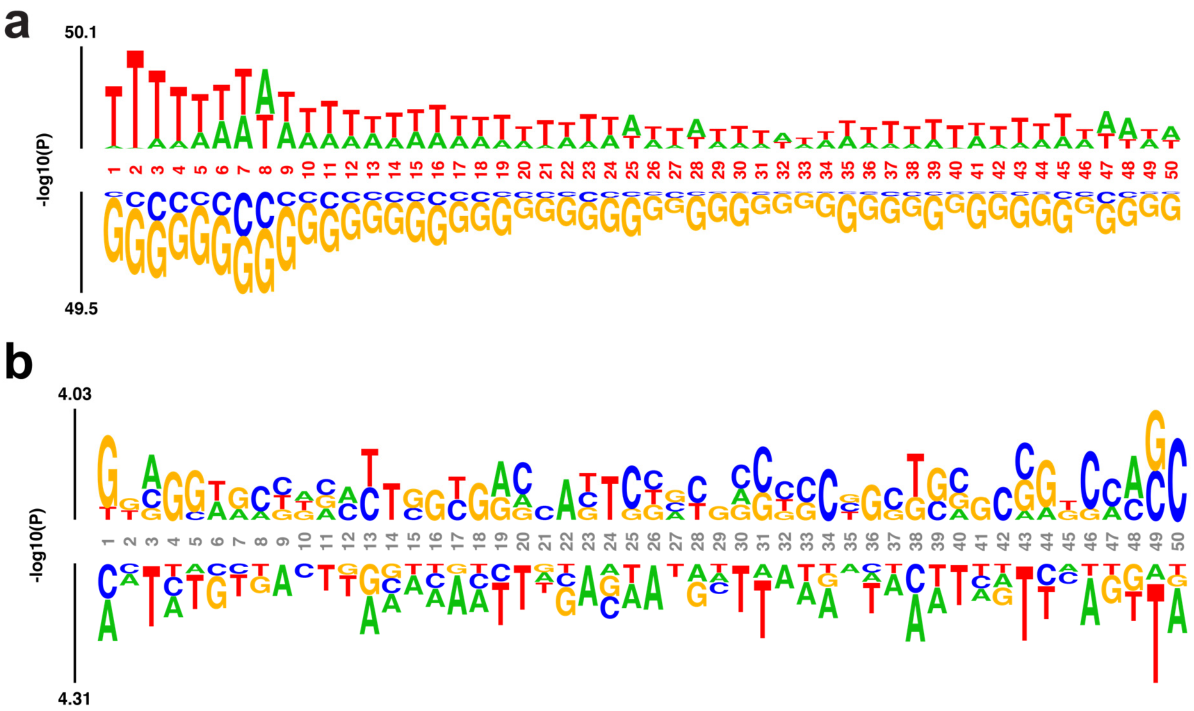

Figure S4. Position-specific enrichment and depletion associated with each base in the N50 region. Probability logo generated by kpLogo for 50,000 random sequences from the **(a)** N50-C and **(b)** N50-EPC libraries. kpLogo weighted sequences using their growth selection enrichment (*Enr*) and searched for *k*-mers of lengths 1-6. Position numbers shown in red indicate enrichment and depletion scores with Bonferroni-corrected p-value < 0.05.

N50-C

+2.5 s.d. (N = 3,357)

Web logo	Occurrences	*p*-value	motif match	motif match *p*-value
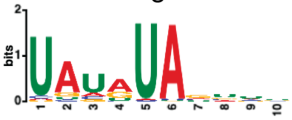	3,079	1.3×10^{-43}	Hrp1	1.1×10^{-4}
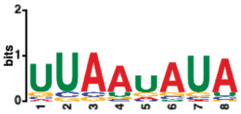	453	0.039	Hrp1	0.040
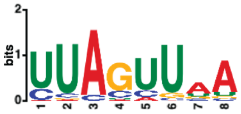	516	0.046	–	–

-2.5 s.d. (N = 8,694)

Web logo	Occurrences	*p*-value	motif match	motif match *p*-value
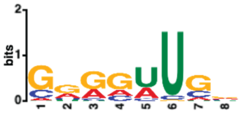	5,910	0.018	–	–

N50-EPC

+2.5 s.d. (N = 1,728)

Web logo	Occurrences	*p*-value	motif match	motif match *p*-value
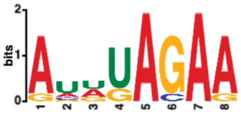	63	0.034	Hrp1	8.2×10^{-3}

-2.5 s.d. (N = 6,617)

Web logo	Occurrences	*p*-value	motif match	motif match *p*-value
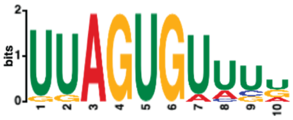	56	0.035	–	–

Figure S5. STREME motif analysis. Statistically significant sequence motifs enriched or depleted in growth selections performed on each library (*Enr* > 2.5 standard deviations above or below the mean) compared against a random subset of sequences from that library. The total numbers of motif occurrences in the library and *p*-value of motif identification by STREME are also shown; also indicated are any significant RNA-binding protein site motif matches from TomTom analysis and their corresponding *p*-values.

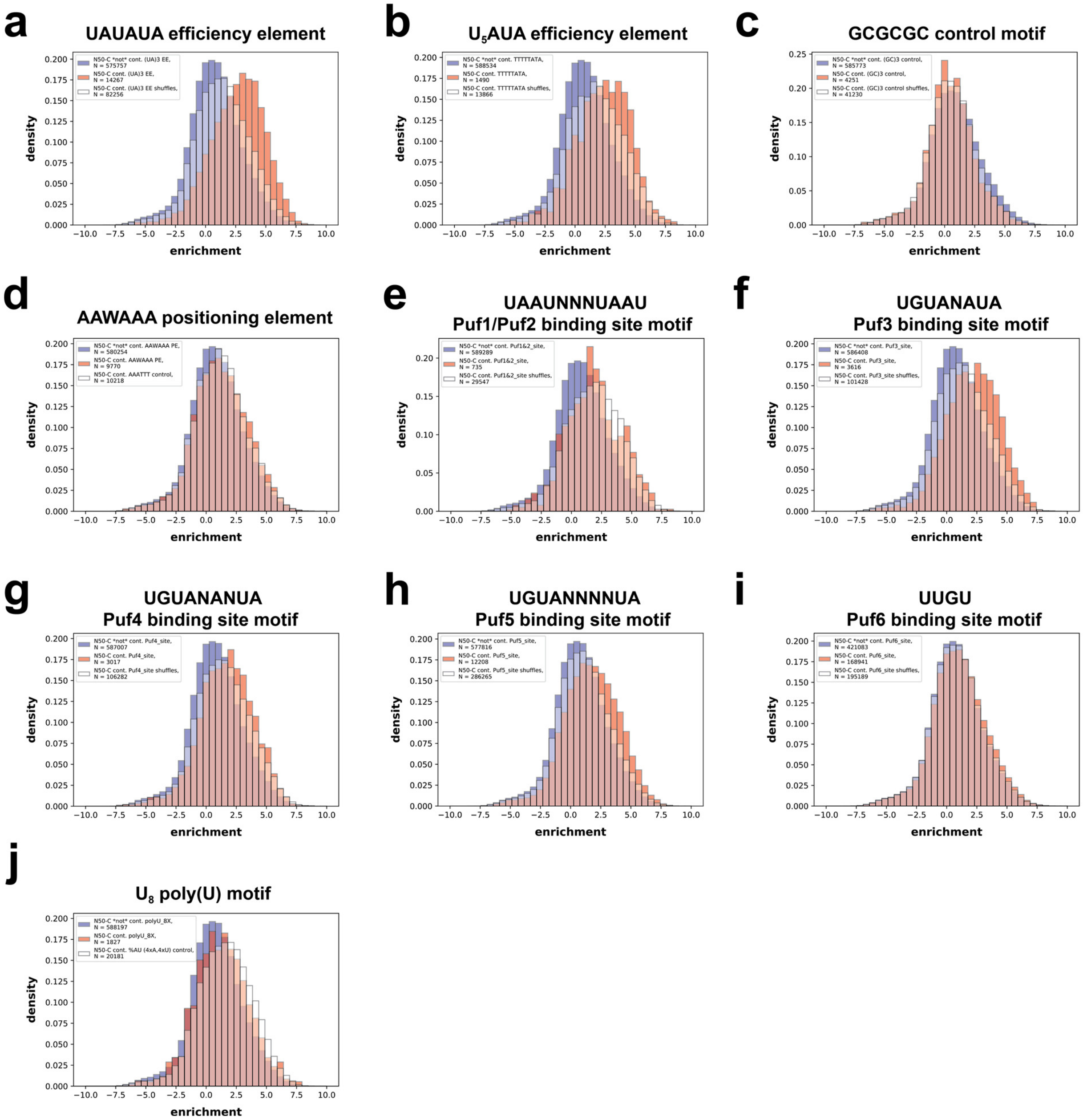

Figure S6. Histograms of 3′ UTR sequence motif effects on growth selection enrichment across the N50-C library, for the indicated motifs. Note that the motifs shown here correspond to those whose mean effects were plotted in Figures 3, 5, 6, 7, and 8. (**a**) – (**j**) Each plot shows the distribution of variant effects for sequences containing the motif in question (red), those lacking this motif (blue), and those containing shuffles of the motif or other control sequences as indicated, but not the motif itself (white).

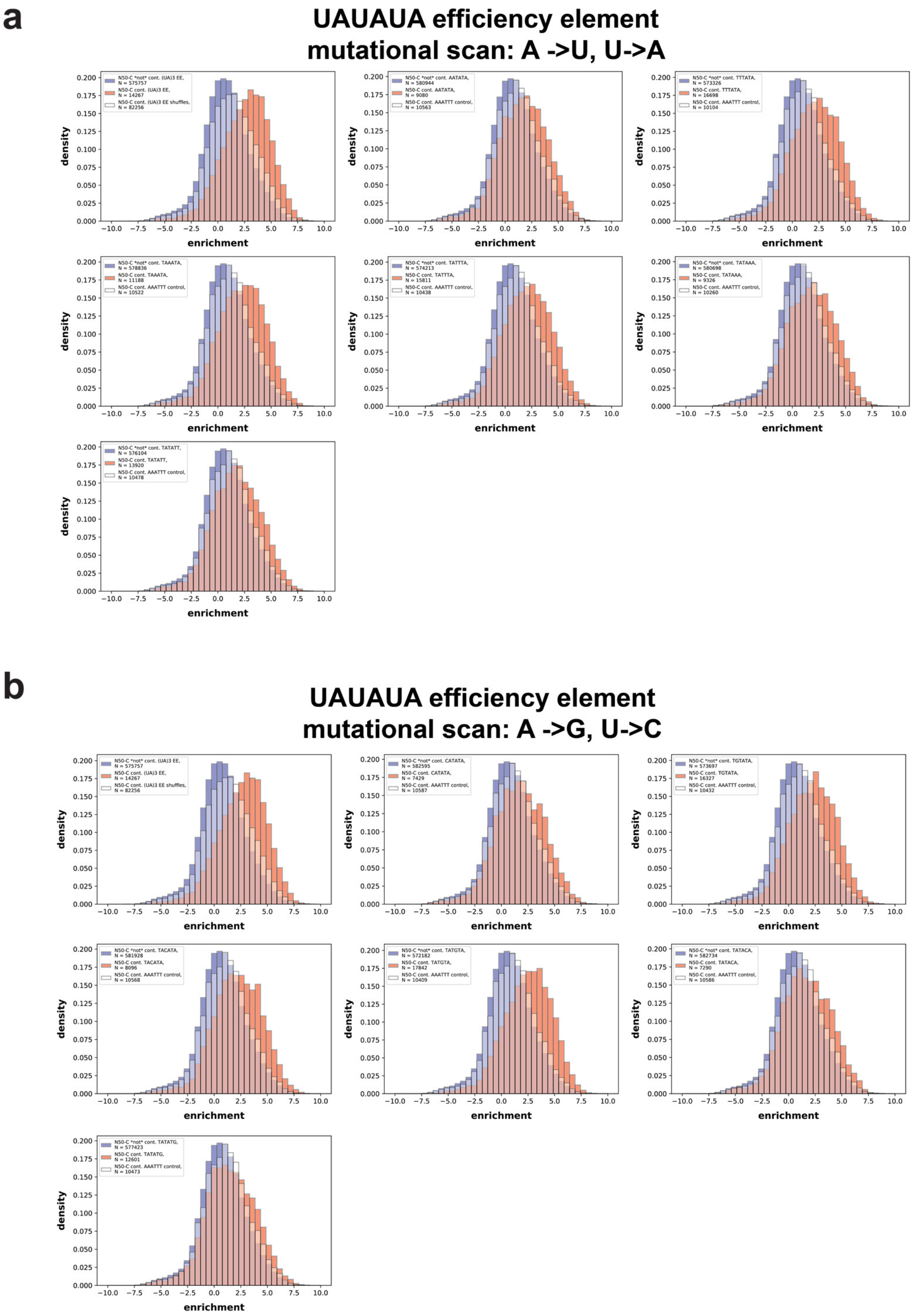

Figure S7, part 1 (continued on next page)

c

U_5AUA efficiency element
mutational scan: A ->U, U->A

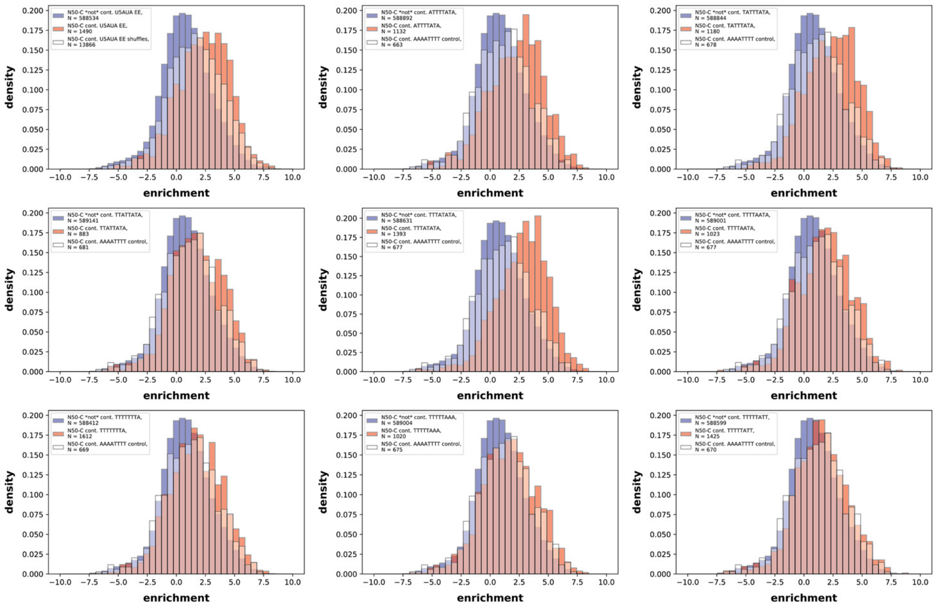

d

$(UA)_4$ motif
mutational scan: A ->U, U->A

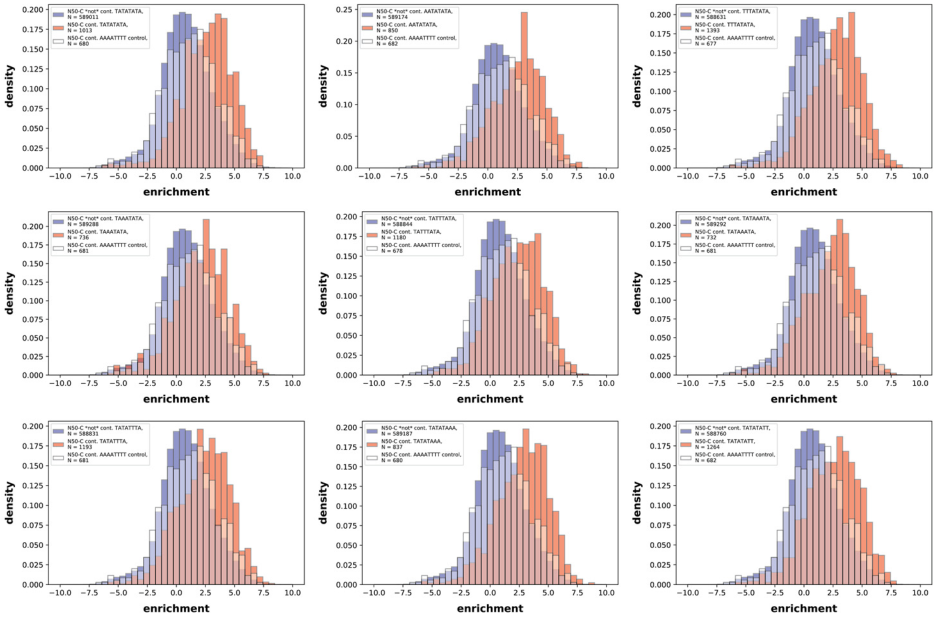

Figure S7, part 2 (continued on next page)

e

$(UA)_5$ motif mutational scan: A ->U, U->A

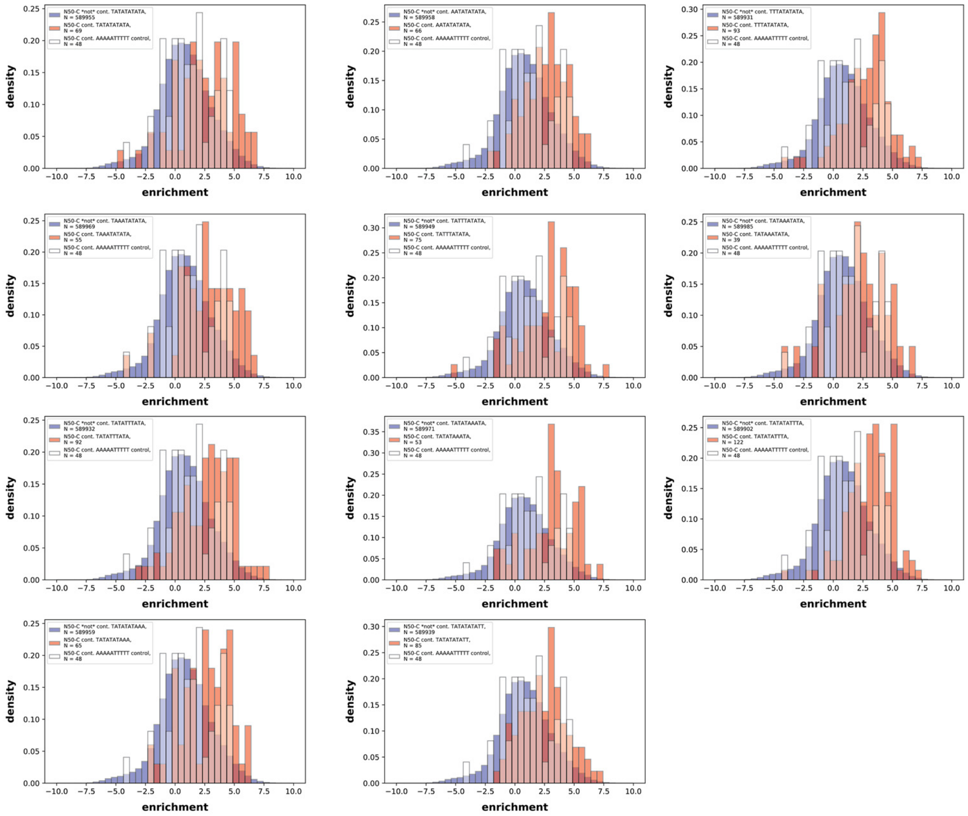

f

AUAUAU motif mutational scan: A ->U, U->A

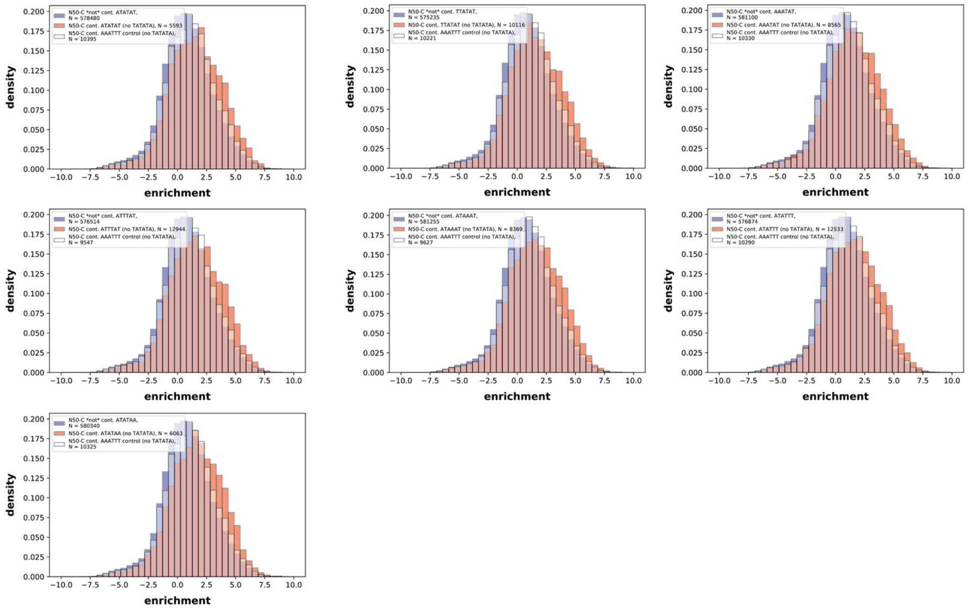

Figure S7, part 3 (caption on next page)

Figure S7. (**a**) – (**f**) Histograms of 3′ UTR sequence motif effects on growth selection enrichment across the N50-C library, for the indicated motifs and their indicated mutant variants, corresponding to the mutational scans of Figure 3. Each plot shows the distribution of variant effects for sequences containing the motif in question (red), those lacking this motif (blue), and those containing shuffles of the motif or other control sequences as indicated, but not the motif itself (white).

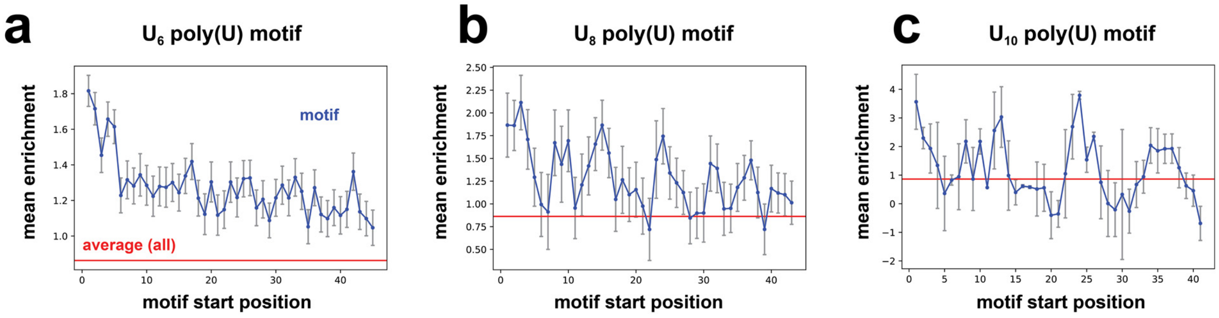

Figure S8. Average per-position growth selection enrichment (as in Figure 3b,d,f) of N50-C 3′ UTR library variants containing poly(U) motifs with 6 (**a**), 8 (**b**), or 10 (**c**) sequential U bases. Blue, sequences containing the motif; red, average enrichment across all N50-C library sequences. Throughout, error bars indicate s.e.m.

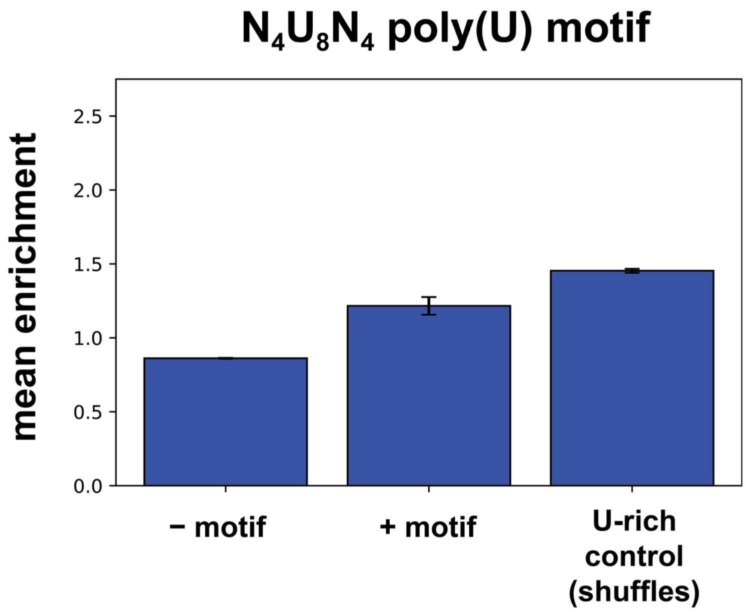

Figure S9. Comparing the gene expression effects of poly(U) sequences with those of equally U-rich sequences. Bars correspond to (left to right): mean enrichment across sequences in the N50-C library lacking $N_4U_8N_4$; mean enrichment across sequences containing $N_4U_8N_4$; and mean enrichment across sequences containing shuffles of $N_4U_8N_4$ with no more than two sequential U's but not $N_4U_8N_4$ itself, while also excluding all sequences containing the consensus efficiency element UAUAUA. Throughout, error bars indicate s.e.m.

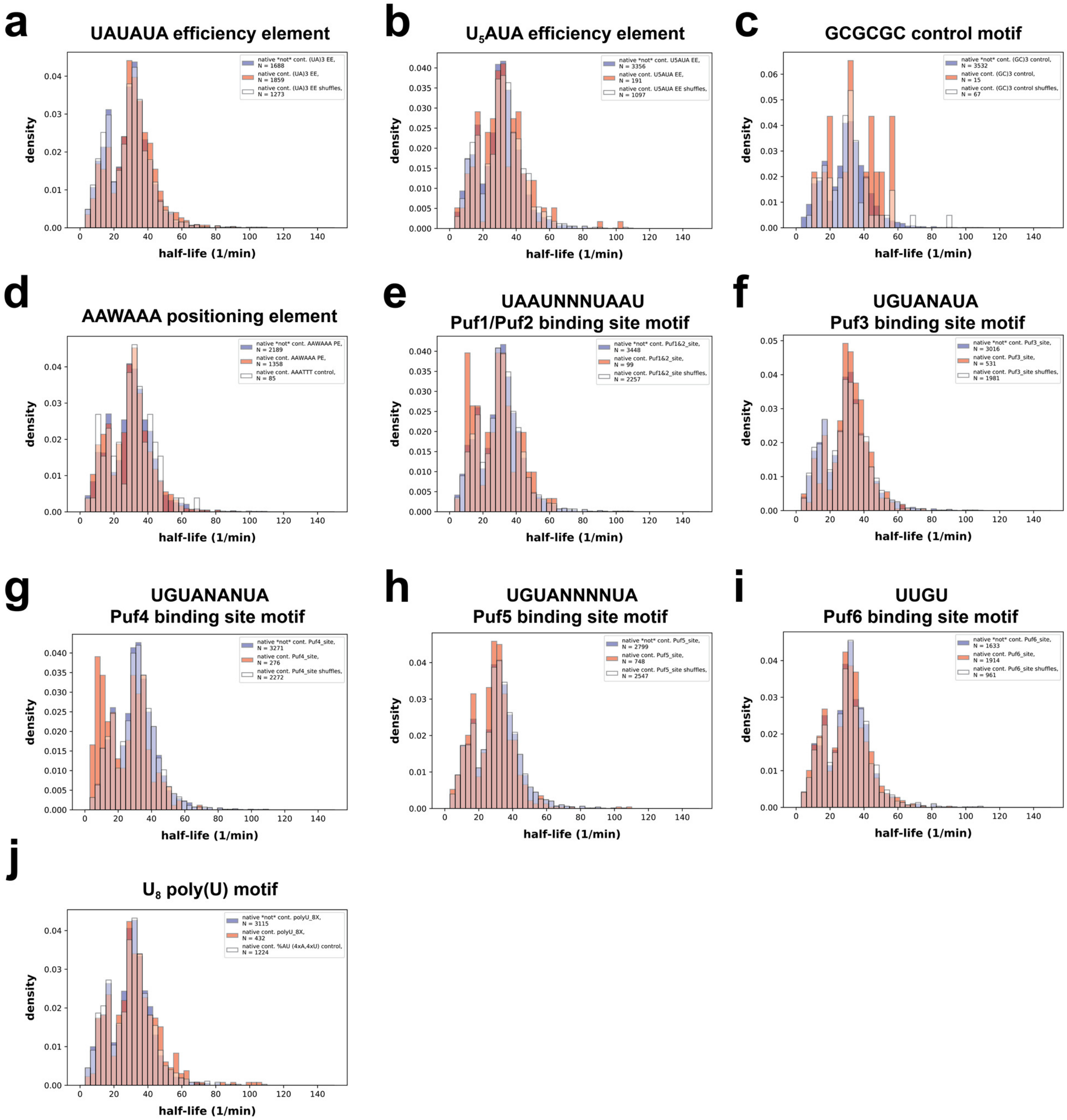

Figure S10. Histograms of 3′ UTR sequence motif effects on mRNA half-life across native yeast genes, based on the data of Geisberg and colleagues [30]. Note that the motifs shown here correspond to those whose mean effects were plotted in Figure 8. (**a**) – (**j**) Each plot shows the distribution of variant effects for sequences containing the motif in question (red), those lacking this motif (blue), and those containing shuffles of the motif or other control sequences as indicated, but not the motif itself (white).

Supplementary Tables

Table S1: Oligonucleotide sequences.

Name	Purpose	Sequence
**Construction of 3′ UTR Libraries**		
F_p415-His	Linearize p415-Cyc1-His3 by inverse PCR	TACAGACGCGTGTACGCATGTAACATTATACTG
R_p415-His	Linearize p415-Cyc1-His3 by inverse PCR	CTACATAAGAACACCTTTGGTGGAGGGAACATC
N50-EPC	Encode 50-EPC library	GTTCCCTCCACCAAAGGTGTTCTTATGTAGNNNNNNNNNNNNNNNNNNNNNNNNNNNNNNNNNNNNNNNNNNNNNNNNNNTATTTATTTTTTTATAGTTATGTTAGTATTAAGAACGTTATTTATATTTCAAATTTTTCTTTTTTTTCTGTACAGACGCGTGTACGCATGTAACATTATA
N50-C	Encode 50-C library	GTTCCCTCCACCAAAGGTGTTCTTATGTAGNNNNNNNNNNNNNNNNNNNNNNNNNNNNNNNNNNNNNNNNNNNNNNNNNNCAAATTTTTCTTTTTTTTCTGTACAGACGCGTGTACGCATGTAACATTATA
F_N50_lib	Amplify N50-EPC and N50-C oligos and make double stranded	TACCAACGATGTTCCCTCCACCAAAGGTGTTCTTATGTAG
R_N50_lib	Amplify N50-EPC and N50-C oligos and make double stranded	AGGTTTTCAGTATAATGTTACATGCGTACACGCGTCTGTA
**Construction of Sequencing Libraries**		
F pri_p415-HIS3_add P5_0	Amplify 3′ UTR for deep sequencing; adds P5 adapter and sequencing index	AATGATACGGCGACCACCGAGATCTACACCATGAGCTGCGGTTGCCATAAGAGAAGC
F pri_p415-HIS3_add P5_2	Amplify 3′ UTR for deep sequencing; adds P5 adapter and sequencing index	AATGATACGGCGACCACCGAGATCTACACGTACATCAGCGGTTGCCATAAGAGAAGC
R pri_p415-HIS3_add P7_0	Amplify 3′ UTR for deep sequencing; adds P7 adapter and sequencing index	CAAGCAGAAGACGGCATACGAGATACATGTCGTATAATGTTACATGCGTACACGCGT
	Amplify 3′ UTR for	CAAGCAGAAGACGGCATACGAGATTAGT

R pri_p415-HIS3_add P7_2	deep sequencing; adds P7 adapter and sequencing index	GCATTATAATGTTACATGCGTACACGCGT
R pri_p415-HIS3_add P7_3	Amplify 3′ UTR for deep sequencing; adds P7 adapter and sequencing index	GCAAGCAGAAGACGGCATACGAGATGCA TACTTATAATGTTACATGCGTACACGCGT
R pri_p415-HIS3_add P7_4	Amplify 3′ UTR for deep sequencing; adds P7 adapter and sequencing index	CAAGCAGAAGACGGCATACGAGATTAGC ATGCTATAATGTTACATGCGTACACGCGT
R pri_p415-HIS3_add P7_5	Amplify 3′ UTR for deep sequencing; adds P7 adapter and sequencing index	CAAGCAGAAGACGGCATACGAGATTTAA GTGTTATAATGTTACATGCGTACACGCGT
R pri_p415-HIS3_add P7_6	Amplify 3′ UTR for deep sequencing; adds P7 adapter and sequencing index	CAAGCAGAAGACGGCATACGAGATGGTT AACTTATAATGTTACATGCGTACACGCGT
Sequencing Read 1	Sequencing primer for Read 1	TACCAACGATGTTCCCTCCACCAAAGGTG TTCTTATGTAG
Sequencing Read 2	Sequencing primer for Read 2	AGAAAAATTTGAAATATAAATAACGTTCT TAATACTAACATAACTATAAAAAAATAAA TA
Sequencing Index 1	Sequencing primer for Index 1	TTTATATTTCAAATTTTTCTTTTTTTTCTGT ACAGACGCGTGTACGCATGTAACATTATA
Sequencing Index 2	Sequencing primer for Index 2	GCTTCTCTTATGGCAACCGC
