# Supplementary material for: Effects of sequence motifs in the yeast 3′ untranslated region determined from massively parallel assays of random sequences
Source: Genome Biol. 2021 Oct 18;22:293. doi: 10.1186/s13059-021-02509-6 (PMC8522215; doi:10.1186/s13059-021-02509-6)
Supplement: Supplementary file 2 — Additional File 2:. Peer review history [file 13059_2021_2509_MOESM2_ESM.docx]

**Review history**

**First round of review**

**Reviewer 1**

Savinov et al. explore the role of sequence elements in the 3′ UTRs of yeast using a large library of random 50mers inserted into a reporter gene 3′ UTR, and measured their relative enrichment after a fitness-based selection.  For some sequence elements, like the efficiency element, their observations agree with studies investigating the elements in their native contexts.  For others, however, their observations are rather different, highlighting the relative important of sequence context for different elements.  Although this study is similar to other yeast 3′ UTR screens (e.g. Shalem et al., PLOS Genetics 2015), this study differs by 1) using a larger variant library, and 2) through the use of a fitness-based enrichment, which permits the continuous measurement of protein output rather than discrete measurements from FACS-defined bins. This study is a somewhat useful resource to yeast post-transcriptional biologists, allowing context- and position-based mutational analyses for thousands of sequence elements.  However, the authors have not exploited their primary data particularly thoroughly, likely missing opportunities to make novel discoveries.  Thus, as currently constructed, the impact of the study is likely to be minimal.  In addition, the authors fail to account for limitations of their screen, which calls into question some of their conclusions.  A revised manuscript, which addresses the issues below, and also extends the analyses, would be more suitable for Genome Biology.

Major points

1.     The authors present no data showing the abundance of 50mer variants within the pool pre- and post-enrichment, which is necessary to show how many times a given variant is being sampled (i.e. the coverage). This is particularly important in a fitness-based screen, which will deplete repressive sequences until they are a small fraction of the pool, or absent completely. Since these repressive sequences get sampled at a much lower frequency, their measurements will be much less precise than those for stabilizing sequences that are more abundant.

2.     The authors fail to correct or acknowledge bias in oligonucleotide synthesis and during PCR amplification of their sequencing libraries, which will skew the pool of 50mers that they investigate. This skewness will change after enrichment, due to major changes in the abundance of each variant (see first major point). This bias could play a role in the enrichment of AU-rich sequences that the authors discuss throughout the manuscript.

3.     The authors state, "We filtered these data to improve our confidence in the input and output variant frequencies…(see Methods)", but this filtering is not described in the Methods at all. It is critical that the authors describe in detail how they filtered the ~3-4 million variants in their plasmid library down to the ~280,000 and ~590,000 post-enrichment variants that were used in all their analyses. This is particularly significant given the first two major points, as a biased filtering at this step will lead to unreliable conclusions.  

4.     There is so much more the authors could (and should) do with the data: additional motifs could be examined, especially novel motifs; could interactions between motifs be considered; are there opportunities to use comparative genomic approaches to extend and validate inferences?

Minor points

-     Figures 3-8: it would be useful to provide the number of variants in each bin to see how many different sequence contexts were sampled for the motif. For example, this information was critical to the interpretation of Fig 8d.

-     Figures 3-8: histograms showing the distribution of variants for a given bin would show more information than simply mean enrichment, and could be critically important for motifs which may behave differently based on sequence context.
o     E.g. the mean enrichment of Puf4 shows that on average it acts as a stabilizing element in the context of random 50mers, but is there a group of 50mers containing Puf4 elements where it acts as a repressive element?

-     Fig 3h: the authors conclude that multiple efficiency elements are detrimental to protein expression because the efficiency element is not enriched in the N50-EPC library (which contains an efficiency element in its constant region). Is this conclusion supported when looking for 50mers containing multiple efficiency elements in the N50-C library?
o     The authors attempt to perform this analysis in Figs 4ef, but look for elongated efficiency elements rather than intact consensus efficiency elements repeated within the 50mer.

-     Fig 6b: the large error bars suggest there are some positions where the Puf1/Puf2 motif acts as a repressive element, could this explain the discrepancy in Fig 8e between your study and published half-lives? See second minor point.

-     Fig 6i: incorrectly labelled as Puf5

-     Considering many N50-C variants lack the efficiency and positioning elements, what fraction of N50-C 3′ UTRs have faithful cleavage at the 3′ cleavage site? Could downstream elements affect the stability of these variants?

-     The absence of references to previous similar publications is perplexing.

**Reviewer 2**

Savinov et al used a reporter assay with a random DNA library to study the effects of 3'UTR sequences on gene expression. The study provides a systematic and unbiased perspective on regulatory elements in 3'UTRs. This perspective allows several new, surprising, and important results about individual regulatory elements, for example the observation that Puf motifs often increase rather than decrease expression. The paper is clearly written and easy to follow.
One overall suggestion for improvement is that the paper should be more transparent in its presentation of statistical tests and the data. See detailed comments below.


Major comments:

1. Did any new motifs emerge beyond those that have been previously described? If not, this should be explicitly mentioned in the Discussion. A major strength of large random DNA libraries is that their unbiased nature can reveal motifs free from preconceptions. As such, an absence of new motifs from this scan is informative about the complexity of expression regulation by the 3'UTR, or, as it were, the apparent absence of such complexity. I'd be keen to read the authors' interpretation of this result.

2. It is not entirely obvious via which selective marker the plasmid library was transformed into yeast. As written, I wondered for a long time whether the selection is via histidine, which would of course confound plasmid presence/copy number with reporter expression. It takes a very detailed read of the Methods section to see that the plasmid selection appears to have been via leucine; this information would be nearly impossible to extract for researchers not working on yeast and thus not well versed in yeast media nomenclature. Please clarify this in the Methods, and maybe also mention that plasmid selection cannot have confounded the readouts in the main text.

3. Was there only one replicate per library? What is the repeatability of the measured effects? If this was not determined, this shortcoming should be clearly stated and the authors should explain why no replicates were performed.

4. All analysis code must be publicly shared with the paper, ideally on Github. This is especially true for the various "custom scripts" in the Methods line 470 to 495 that represent the core of the enrichment and motif analyses.

5. Line 471: How were standard errors computed?

6. Figure 2 C & D, and text lines 127 & 128: why are all (!) the log2 enrichments for the N50-C library positive, and all the enrichments for the N50-EPC library negative? Shouldn't these be centered on zero in each library, perhaps with a compressed range in EPC if the sequences matter less there? How can every k-mer be positively enriched in N50-C? Doesn't there have to be a set of k-mers that are underrepresented in more highly expressed sequences to balance all these positive enrichments? Why is GGGGGG still positively enriched, indicating it drives higher expression compared to other sequences? I feel I am missing a crucial bit of information about how these enrichments are calculated. Please clarify this in the Results, the Methods, and/or the figure legend.

7. Figures 3 to 8: the barplots used to display the data hide important information about the data distributions. Specifically, they only show the mean and (presumably, this is not stated) the standard error. The authors themselves note at various points that each of these bars contains a range of values; this information is invisible as displayed. Please replace all bar plots with displays that allow the reader to see and interpret the distribution of the data. Box plots or violin plots would be much more informative.

8. Please add the  p-value for the comparisons described starting in line 181, as well as for the matched comparison in the N50-C library. Alternatively, these p-values could be added to Figure 3 g & h.

9. Page 13 bottom paragraph on Puf proteins: it is unclear if "significantly" in this section refers to significant p-values or a more vague notion of biological significance. From Figure 6, and assuming the error bars are standard errors, Puf1/2 may still have statistically significant but small effects. Please clarify what "significant" means here, and please add p-values to these comparisons, in particular if the claim is that motifs "did not significantly impact" expression (line 263).

10. In all figures, state in the legends what do the various error bars indicate. Standard errors of the mean? Ranges? Quartiles?

11. Line 336: state the p-value for this non-significant difference. In fact, for consistency, p-values need to be added to all of the comparisons that take the form of this analysis (plus/minus motif and shuffle, Figures 3 to 8). They can be easily added to the figures without cluttering the text. Also see my comment above about replacing the bar plots with more informative displays.

12. A difference "within one s.e.m." is sometimes "only a weak nominal reduction" (Line 355) and other times "not significant" (line 336). Which is it? This issue will be avoided by adding p-values throughout (see comment above).


Minor comments:

13. Figure 2 A & B: please provide p-values for the two correlation coefficients

14. Line 160: "The effects of shuffled hexamers derived from UAUAUA showed little position-dependence". The green line does look higher on the left than on the right, so it would be more conservative to say "...showed less position dependence than the motif, [...]"

15. It would add visual clarity if Figure 3 panels b,d,f were on the same y-axis scale.

16. Line 126: To clarify why the "average" enrichment across 6-mers has a range, I'd change this sentence to "We found that the average log2 enrichments of random sequences containing the given 6mer ranged..."

17. Line 201/202 "with this variant being the second highest-ranked hexamer sequence (Fig. 2c).". This variant is not currently indicated in Figure 2c. Likewise for line 211.

18. Figure 6i is mistitled: should be Puf6, not Puf5

**Authors’ response to reviewers**

**Reviewer #1:**

Major points

1. The authors present no data showing the abundance of 50mer variants within the pool pre- and post-enrichment, which is necessary to show how many times a given variant is being sampled (i.e. the coverage). This is particularly important in a fitness-based screen, which will deplete repressive sequences until they are a small fraction of the pool, or absent completely. Since these repressive sequences get sampled at a much lower frequency, their measurements will be much less precise than those for stabilizing sequences that are more abundant.

**As noted in the “Availability of data and materials” section, we include with the revised manuscript a supplementary Source Data file containing information on each sequence variant analyzed, including the frequencies (abundance), read counts and enrichments of each variant. We controlled for precision in enrichment measurements by imposing a quality filter based on minimum read counts (described in our response to Reviewer #1 major point 3).**

**Additionally, as the manuscript is focused on the average effects of sequence elements in a random sequence context, across many random N50 sequences, none of its conclusions depend on the precision of enrichment determined for an individual library sequence. Many random sequences containing the motif of interest provide the background, which buffers the results against potential issues with the precision of individual sequence variant effects (see also our response to Reviewer #2 major comment 3).**

2. The authors fail to correct or acknowledge bias in oligonucleotide synthesis and during PCR amplification of their sequencing libraries, which will skew the pool of 50mers that they investigate. This skewness will change after enrichment, due to major changes in the abundance of each variant (see first major point). This bias could play a role in the enrichment of AU-rich sequences that the authors discuss throughout the manuscript.

**Although there are known biases in oligonucleotide synthesis and in PCR amplification that would result in over-representation of some sequences in the input library, these biases should not affect the enrichment scores for any individual sequence, as these scores are determined by the change in frequency during selection (after the synthesis step). Any such bias during PCR amplification should affect growth selection input and output sequences equally.**

**The observed enrichment of AU-rich sequences is consistent with other published observations that AU-rich 3′ UTR sequences lead to higher levels of protein production (*e.g.*, Rabani *et al.* (2017) *Mol. Cell* 68, 1083-1094; Goodman *et al.* (2013) *Science* 342, 475-479; Litterman *et al.* (2019) *Genome Res.* 29, 896-906). Thus, the strong enrichment of AU-rich sequences might be expected from the existing literature.**

3. The authors state, "We filtered these data to improve our confidence in the input and output variant frequencies…(see Methods)", but this filtering is not described in the Methods at all. It is critical that the authors describe in detail how they filtered the ~3-4 million variants in their plasmid library down to the

~280,000 and ~590,000 post-enrichment variants that were used in all their analyses. This is particularly significant given the first two major points, as a biased filtering at this step will lead to unreliable conclusions.

**We clarified how quality-filtering of sequence variants was performed. In all analyses, we considered only sequences with at least 5 reads in the selection input, and at least 1 read in the selection output (no pseudocounting was performed). In the revised manuscript, this filtering is now described in detail in the Methods (p. 27) and the general approach (filtering based on minimum read counts) is provided in the main text (p. 7).**

4. There is so much more the authors could (and should) do with the data: additional motifs could be examined, especially novel motifs; could interactions between motifs be considered; are there opportunities to use comparative genomic approaches to extend and validate inferences?

**We performed extensive additional analyses in response to this reviewer suggestion, resulting in 5 new figures and accompanying text (pp. 9-10), as described below. Comprehensive data tables as well as associated statistics (s.e.m., *N*) have also been included in the Source Data.**

**In addition to our analyses of the average protein expression effects of each possible 6-mer, included in the original manuscript, we have expanded this *k*-mer analysis to include every possible 4-, 5-, 7-, and 8-mer for both libraries (Supplementary Figures 1 and 3). The results from *k*-mer analyses of the N50-C library demonstrate that (UA)_N_ sequences and their point mutants produce the highest levels of expression, and from the N50-EPC library results, enrichment is generally insensitive to *k*- mer content. To analyze the *k*-mer results further, we examined AU content of *k*-mers and the associated growth selection enrichment in both libraries (Supplementary Figure 2). AU content of *k*- mers was correlated with enrichment across 4–8-mers in the N50-C library, but not in the N50-EPC library. These findings are consistent with our determination that (UA)_N_ sequences are the major drivers of expression in the N50-C context, whereas the constant presence of the three known elements (efficiency, positioning, and cleavage) in the N50-EPC library dominates expression.**

**To further interrogate the data, we determined position-specific enrichment and depletion of bases across the N50 sequences in both libraries using kpLogo (Supplementary Figure 4). These results demonstrate that U and A bases are associated with enrichment throughout the N50 sequence, and especially near the 5′ end; G bases are associated with depletion throughout the sequence. In contrast, in the N50-EPC library, no base was significantly associated with enrichment or depletion at any N50 position. These results support the *k*-mer analyses: in the N50-C library, AU-rich and especially (UA)_N_ features drive high expression while stretches of G bases are associated with low expression, but no trends hold for the N50-EPC library.**

**We also looked for new motifs associated with either high or low enrichment by searching for new sequence elements *de novo* using the MEME Suite program STREME. The most significant motif discovered by STREME was a motif in the N50-C library associated with enrichment and containing a UAUAUA (consensus efficiency element) sequence. The few other motifs output by this analysis had *p*-values that were barely significant (close to 0.05), especially considering the large size of our libraries. TomTom analysis to find known RNA-binding protein sites matching the STREME- discovered motifs found significant hits only for the Hrp1 protein, which binds the efficiency element. Other STREME-discovered motifs (again barely significant) had no significant match to known RNA-binding protein sites (Supplementary Figure 5).**

**These analyses suggest that efficiency elements are the main drivers of protein expression levels in yeast 3′ UTRs, consistent with other work (*e.g.* Shalem *et al.*, *PloS Genetics* 2015). We show that this phenomenon holds in a random sequence context, not just among native yeast genes. Perhaps, in part due to the strong effects of efficiency elements, it is difficult to detect the effects of other motifs in these experiments, again consistent with prior studies.**

**While the original submission contained a consideration of motif interactions between the efficiency element and positioning elements (Fig. 5), we now also consider the effects of multiple efficiency elements in both the N50-EPC and N50-C contexts (pp. 12-13). With this random library design, co- occurrence of motifs is rare, preventing a more systematic study of interactions. Further consideration of this question is outside the scope of the current work, but would be an interesting avenue for future studies.**

Minor points

- Figures 3-8: it would be useful to provide the number of variants in each bin to see how many different sequence contexts were sampled for the motif. For example, this information was critical to the interpretation of Fig 8d.

**The number of variants lacking or containing various sequence motifs (or their associated control sequences) has been added throughout the figure legends, and such variant counts have been extensively documented in the Source Data.**

- Figures 3-8: histograms showing the distribution of variants for a given bin would show more information than simply mean enrichment, and could be critically important for motifs which may behave differently based on sequence context.
- E.g. the mean enrichment of Puf4 shows that on average it acts as a stabilizing element in the context of random 50mers, but is there a group of 50mers containing Puf4 elements where it acts as a repressive element?

**In the relevant plots throughout the manuscript, we sought to demonstrate the mean effects of 3′ UTR motifs on gene expression in a random sequence context. As this is a diverse random library by design, the distributions of effects are broad; conclusions specifically about mean effects would be**

**harder to discern in a different representation of the data also showing the distribution of motif effects. Therefore, to maintain clarity, we have not altered the way these data are represented in the main text figures.**

**However, in response to the reviewer suggestion (and the similar comment 7 by Reviewer #2), we now include with the revised manuscript an extensive set of histograms showing the distributions of effects of variants lacking or carrying each sequence motif (or its corresponding control sequence) as figure panels in the new Supplementary Figures 6, 7 and 10. These supplementary figures are referred to in both the text and the associated main text figure legends such that the distributions of motif effects on expression can be readily located.**

- Fig 3h: the authors conclude that multiple efficiency elements are detrimental to protein expression because the efficiency element is not enriched in the N50-EPC library (which contains an efficiency element in its constant region). Is this conclusion supported when looking for 50mers containing multiple efficiency elements in the N50-C library?
- The authors attempt to perform this analysis in Figs 4ef, but look for elongated efficiency elements rather than intact consensus efficiency elements repeated within the 50mer.

**Upon the reviewer’s suggestion, we performed an analysis of the effects of multiple (non-overlapping) consensus efficiency elements ((UA)_3_ motifs) in the N50-C library context. Although sparsely represented in the library, N50-C sequences with two consensus efficiency elements (*N* = 81) have significantly higher protein expression on average (<*Enr*> = 3.38 +/- 0.23) than sequences with a single consensus efficiency element (*N* = 14,186, <*Enr*> = 2.60 +/- 0.03), in contrast to the N50-EPC results. Therefore, our results regarding slightly detrimental effects in the N50-EPC context are specific to the optimized context rather than a generic feature of efficiency elements in 3′ UTRs. These results and corresponding discussion have been added to the revised manuscript (pp. 12-13).**

**Given these findings, we removed the sentence at lines 223-225 of the original manuscript interpreting our results for elongated efficiency elements ((UA)_N_) as supporting a “threshold model” for UTR function. We replaced this sentence with one discussing how these results suggest that longer (UA)_N_ sequences do not yield improved Hrp1-binding activity compared to (UA)_3_ (pp. 14-15).**

- Fig 6b: the large error bars suggest there are some positions where the Puf1/Puf2 motif acts as a repressive element, could this explain the discrepancy in Fig 8e between your study and published half- lives? See second minor point.

**The relatively large s.e.m. (error bars) for positional effects of Puf1/Puf2 binding site motifs are not unique to this sequence element; we generally observe similarly large errors for positional effects of most elements, including, for example, the alternative efficiency element U_5_AUA (Fig. 3d). These generally large error bars are strongly influenced by the low-*N* statistics of element start site occurrence at each residue of the random 50-mer (N50), as expected in this large random library (e.g., average *N* ~ 15 at any given sequence position for the Puf1/Puf2 site motif). Thus, although it is possible that there are some 3′ UTR positions where a subset of Puf1/Puf2 binding sites act as**

**repressive elements in our library, the relatively large error bars for Fig. 6b do not provide evidence for such an effect.**

**The nature of the library (designed to be as diverse as possible), combined with the relatively sparse sequence counts for motifs initiating at each N50 position, preclude analysis of the distribution of sequences with motifs starting at each position. Thus, we do not attempt to draw conclusions about subsets of motifs located at specific positions in the 50-mer sequence. However, the reviewer’s question is an interesting one; exploration of this question with tailored sequence libraries with more coverage of motifs of interest at particular 3’ UTR sequence positions would be a direction for future studies.**

- Fig 6i: incorrectly labelled as Puf5

**We corrected the label for this figure panel.**

- Considering many N50-C variants lack the efficiency and positioning elements, what fraction of N50-C 3′ UTRs have faithful cleavage at the 3′ cleavage site? Could downstream elements affect the stability of these variants?

**The reviewer raises an interesting question, but one that is not readily addressable using our approach, as RNA-seq is challenging to perform on such a diverse random library because of the sheer number of different 3′ UTR construct variants and their differing levels of transcription. Even with targeted RNA-seq, reasonably sampling the library at the mRNA level is a challenge, due to sampling from a pool of RNA which is massive compared to the levels of individual variant mRNAs from the library. Indeed, we did attempt RNA sequencing to address questions such as this one, but were only able to sparsely sample the library, with clear bottleneck effects. Thus, we did not proceed further with this line of work.**

- The absence of references to previous similar publications is perplexing.

**We now cite an additional nine papers (reference numbers 7, 9-16) in the Introduction.**

**Reviewer #2**:

Major comments:

1. Did any new motifs emerge beyond those that have been previously described? If not, this should be explicitly mentioned in the Discussion. A major strength of large random DNA libraries is that their unbiased nature can reveal motifs free from preconceptions. As such, an absence of new motifs from this scan is informative about the complexity of expression regulation by the 3′UTR, or, as it were, the apparent absence of such complexity. I'd be keen to read the authors' interpretation of this result.

**Please see our response to Reviewer #1 major comment 4.**

2. It is not entirely obvious via which selective marker the plasmid library was transformed into yeast. As written, I wondered for a long time whether the selection is via histidine, which would of course confound plasmid presence/copy number with reporter expression. It takes a very detailed read of the Methods section to see that the plasmid selection appears to have been via leucine; this information would be nearly impossible to extract for researchers not working on yeast and thus not well versed in yeast media nomenclature. Please clarify this in the Methods, and maybe also mention that plasmid selection cannot have confounded the readouts in the main text.

**We clarified the selectable marker used to maintain the library in yeast (*LEU2*), and pointed out that it is distinct from the plasmid-borne *HIS3* gene used for growth selection (main text, p. 5, 6 and Methods, p. 23, 24).**

3. Was there only one replicate per library? What is the repeatability of the measured effects? If this was not determined, this shortcoming should be clearly stated and the authors should explain why no replicates were performed.

**A single replicate per library was performed for these experiments, as now indicated (main text, p. 7; Methods, p. 26). We explain that the size of these experiments and random nature of the variant library preclude true biological replicates (it is not possible to independently reintroduce variants into cells because a different set of random sequences would be selected during cloning and/or transformation). There is precedent for these types of assays to be reported for single replicates (e.g. Rosenberg *et al.*, *Cell* 2015; Cuperus *et al.*, *Genome Res.* 2017; Sample *et al.*, *Nat. Biotech.* 2019; De Boer *et al.*, *Nat. Biotech.* 2020).**

**However, an alternative measure of reproducibility for these measurements can be used. Analyses of the effects of specific sequence motifs in a random sequence context across many – typically thousands – of diverse N50 sequences carrying a given element (rather than the specific enrichment of any single library sequence variant) provide an approximation of a generic random sequence background for each element of interest. Reproducibility of the effects of sequence features can thus be determined from the mean ± s.e.m. effects of each sequence element across the library, as reported throughout the figures (see also the Source Data).**

4. All analysis code must be publicly shared with the paper, ideally on Github. This is especially true for the various "custom scripts" in the Methods line 470 to 495 that represent the core of the enrichment and motif analyses.

**Analysis scripts have now been shared via GitHub, with a Zenodo-generated permanent doi linking to the code provided in the manuscript (see “Availability of data and materials”).**

5. Line 471: How were standard errors computed?

**Standard errors for each subset of variants (*e.g.*, those containing a motif of interest) were calculated as the standard deviation divided by the square root of variant counts (described on p. 27).**

6. Figure 2 C & D, and text lines 127 & 128: why are all (!) the log2 enrichments for the N50-C library positive, and all the enrichments for the N50-EPC library negative? Shouldn't these be centered on zero in each library, perhaps with a compressed range in EPC if the sequences matter less there? How can every k- mer be positively enriched in N50-C? Doesn't there have to be a set of k-mers that are underrepresented in more highly expressed sequences to balance all these positive enrichments? Why is GGGGGG still positively enriched, indicating it drives higher expression compared to other sequences? I feel I am missing a crucial bit of information about how these enrichments are calculated. Please clarify this in the Results, the Methods, and/or the figure legend.

**We thank the reviewer for pointing out this potential point of confusion. Below and in the revised manuscript, we provide clarification.**

**To begin with, not all sequence variants gave positive enrichments for the N50-C library, and not all sequence variants gave negative enrichments in the N50-EPC library; both libraries contained a range of depleted and enriched variants. However, as the reviewer points out, the average enrichment of every possible 6-mer sequence is indeed positive for the N50-C library, and negative for the N50- EPC library.**

**The confusion derives from the nature of massively-parallel competitions between cells carrying different sequences to grow and divide in a complex population. In such competitions, the reference point for enrichment or depletion in the selection is somewhat arbitrary, as the ways in which the population frequencies of sequences change depend on the structure of the population. For example, a library of distinct sequences all producing equal, high growth rates would yield all sequences having an enrichment of roughly zero, because no sequence would have a competitive advantage relative to another. On the other hand, sequences producing this same high growth rate would yield a positive enrichment in the context of a library containing many sequences producing lower growth rates. Therefore, the enrichment of a variant as positive or negative is not particularly informative on its own; some reference point is needed. Throughout the manuscript, the reference point we choose is the average enrichment of all sequences in the library (or almost equivalently given the size and randomness of the libraries, the average enrichment of all sequences lacking a given motif). This reference point is depicted as a red line in Figure 2c-d. Individual random 50-mers and average effects of sequence motifs can then be fruitfully compared by considering effects relative to the library average, as we do throughout the paper (e.g., GGGGGG is depleted relative to the mean enrichment for the library).**

**In response to the reviewer’s comment, we added to Figure 2c-d a second reference point for comparing motif effects. Both libraries contained a shared sequence producing a known high growth rate: the construct bearing an intact, unaltered *CYC1* 3′ UTR (no N50 sequence inserted). We added this reference enrichment value to both figure panels as an orange line (and the enrichment values**

**are noted in the main text). The wild type *CYC1* 3′ UTR provides a positive enrichment in the N50-C library (2.25) and in the N50-EPC library (0.73). When the library mean enrichment is normalized to the wild type *CYC1* 3′ UTR enrichment, the normalized mean enrichments are almost identical between the two libraries (-1.39 vs. -1.31), as might be expected. These plots and calculations demonstrate that the average random N50 sequence is depleted compared to the wild type *CYC1* in both libraries, and that the extent of relative depletion is similar for the two libraries. We explain these considerations in the main text (pp. 7-8).**

7. Figures 3 to 8: the barplots used to display the data hide important information about the data distributions. Specifically, they only show the mean and (presumably, this is not stated) the standard error. The authors themselves note at various points that each of these bars contains a range of values; this information is invisible as displayed. Please replace all bar plots with displays that allow the reader to see and interpret the distribution of the data. Box plots or violin plots would be much more informative.

**The error bars indicate standard error of the mean in all figures (see also response to Reviewer #2 major comment 10), as is now stated in all figure legends.**

**With regard to the representation of the data in figures 3 - 8, please see our response to Reviewer #1 minor comment 2. We include with the revised manuscript an extensive set of histograms showing the distributions of effects of variants lacking or carrying each sequence motif (or its corresponding control sequence) as figure panels in the new Supplementary Figures 6, 7 and 10.**

8. Please add the p-value for the comparisons described starting in line 181, as well as for the matched comparison in the N50-C library. Alternatively, these p-values could be added to Figure 3 g & h.

**We added *p*-values for comparisons of variants lacking or containing various sequence motifs (or containing appropriate control sequences, e.g., shuffles of the motif) throughout the figures, including Figure 3 panels g and h. See also our response to Reviewer #2 comments 9 and 11.**

9. Page 13 bottom paragraph on Puf proteins: it is unclear if "significantly" in this section refers to significant p-values or a more vague notion of biological significance. From Figure 6, and assuming the error bars are standard errors, Puf1/2 may still have statistically significant but small effects. Please clarify what "significant" means here, and please add p-values to these comparisons, in particular if the claim is that motifs "did not significantly impact" expression (line 263).

**Error bars indeed indicate standard errors throughout the figures. As noted in the response to Reviewer #2 comment 8, we added *p*-values for comparisons of variants lacking or containing various sequence motifs (or containing appropriate control sequences, such as shuffles of the motif) throughout the figures, including Figure 6. The Puf1/2 binding sites have no statistically significant effects on His3 protein expression levels (as reflected by the growth selection enrichment) compared**

**to shuffled control sequences. Given the added *p*-values, we believe the original text is appropriate for this comparison.**

10. In all figures, state in the legends what do the various error bars indicate. Standard errors of the mean? Ranges? Quartiles?

**Error bars were previously described as standard errors, but only in Figure 2. We now state in all figure legends that the error bars indicate standard errors of the mean.**

11. Line 336: state the p-value for this non-significant difference. In fact, for consistency, p-values need to be added to all of the comparisons that take the form of this analysis (plus/minus motif and shuffle, Figures 3 to 8). They can be easily added to the figures without cluttering the text. Also see my comment above about replacing the bar plots with more informative displays.

**As stated above, we added *p*-values throughout the figures, including Figure 8e. For the comparison at line 412 (originally line 336) we clarify that the effect size is not statistically significant.**

**As discussed in the response to Reviewer #2 major comment 7 and Reviewer #1 minor comment 2, we supplemented the main text figures showing the essential results (mean effects ± s.e.m.) with an extensive set of histograms showing the distribution of effects of each motif and relevant controls (in the new Supplementary Figures 6, 7, and 10.**

12. A difference "within one s.e.m." is sometimes "only a weak nominal reduction" (Line 355) and other times "not significant" (line 336). Which is it? This issue will be avoided by adding p-values throughout (see comment above).

**We discussed the weak nominal reduction (original line 355) to comment on this difference in qualitative terms without claiming that it is statistically significant. However, this has been clarified in the revised manuscript as suggested; as noted in the responses above we systematically added *p*- values throughout the manuscript. We additionally clarified our language around the magnitudes and significance of motif effects to reflect the results of these statistical tests, including at lines 431- 432 and 412 (originally lines 355 and 336).**

Minor comments:

13. Figure 2 A & B: please provide p-values for the two correlation coefficients

**We provided *p*-values for these correlations in the figure legends for Fig. 2a-b. Given the vast number of datapoints involved, even the weak correlation in panel b is highly statistically significant.**

14. Line 160: "The effects of shuffled hexamers derived from UAUAUA showed little position- dependence". The green line does look higher on the left than on the right, so it would be more conservative to say "...showed less position dependence than the motif, [...]"

**Although “little position-dependence” was not meant to imply a lack of position-dependence, we altered the sentence in question in the spirit of the reviewer’s suggestion, to avoid any such implication (revised manuscript line 226).**

15. It would add visual clarity if Figure 3 panels b,d,f were on the same y-axis scale.

**We made this change.**

16. Line 126: To clarify why the "average" enrichment across 6-mers has a range, I'd change this sentence to "We found that the average log2 enrichments of random sequences containing the given 6mer ranged..."

**As suggested, we edited this sentence for enhanced clarity regarding the range of average 6-mer effects (revised manuscript line 133).**

17. Line 201/202 "with this variant being the second highest-ranked hexamer sequence (Fig. 2c).". This variant is not currently indicated in Figure 2c. Likewise for line 211.

**The variants noted at original manuscript lines 201-202 and 211 (revised manuscript lines 272-273 and 281-282) are now indicated in the revised Figure 2c.**

18. Figure 6i is mistitled: should be Puf6, not Puf5

**This has been corrected in the revised manuscript.**

**Reviewer #3**:

I have only very minor suggestions/questions:

1. They report 590,000 quantified sequences for the N50-C library, which is an impressive number for an MPRA in yeast, but also a very small subsample of all possible 50 nt sequences. Given that, it would be useful to know how well each of the possible 6-mers or other motifs analyzed are covered by the 50 nt library. For example, in figure 5C looking at co-occurrence of EE and PE elements, what is the N of each bin? For each possible k-mer figure 2 c and d, how many times, on average was each 6-mer represented in the quantified library.

**In the revised manuscript, we now provide a Source Data file containing various statistics for the set of variants containing each 6-mer sequence, including the mean enrichment and its standard error, as well as the number of variants (*N*) in the library containing that sequence. We also now note in the main text of the manuscript the minimum and typical (mean ± s.d.) number of sequence variants containing any given hexamer sequence in the N50-C library (p. 8). Overall, we note that most motifs investigated are covered by thousands of sequences in the library. Similarly, we provide values of *N* for bar plots throughout the figure legends, including Figure 5C (in this case motif co-occurrence is limited compared to individual motifs; see also response to minor comment 2 below, and Reviewer #1 minor point 3 regarding sequences containing two consensus efficiency elements).**

2. Relatedly, was there enough coverage of sequence space to search for additional motif combinations, or for context elements that might explain the discrepancy, for example, between the strong effect of the efficiency element in the N50 library versus the native 3′ UTRs? Are there enriched motifs in the enhancing versus repressing occurrences of the Puf3 motif in the library? Most of the analysis of the paper is focused on short motifs, but one of the conclusions is that context matters. Is it possible to find important contextual elements in the library.

**The reviewer raises an interesting question. However, as noted, motif combinations are typically covered sparsely. For example, the UAUAUA element has *N* ~14,000, yet only 81 N50-C sequences contain two (non-overlapping) instances of this motif. See our response to Reviewer #1 minor point 4 regarding distributions of sequence variants containing motifs at each position in the N50. As we note in response to point 1, the *N* for different bins of sequences containing both an efficiency and positioning element were low as well. Even for single motifs such as the Puf3 binding site, the distributions of effects across the random library are broad yet unimodal, not providing an obvious way to search for context effects; the same considerations as for pairs of motifs apply to motifs appearing in a given context, e.g. flanked by particular types of sequences. Because of the limited statistics due to sparse sampling, we did not further analyze contextual effects or motif combinations.**

Finally, we note that in the process of revising the manuscript, we found that the mRNA half-life data (green bars) in panel (a) of Figure 8 of the original submission were mistakenly based on an outdated version of our analysis. No other figure panel (or any other part of the manuscript) was affected by this issue. We corrected Figure 8a with the updated calculations in the revised manuscript. This correction did not change our interpretation of the data in the manuscript text.

**Second round of review**

**Reviewer 1**

The authors have made significant and valuable changes to the manuscript based on reviewer comments, in particular the inclusion of histograms to show the distribution of motifs, the inclusion of sequence abundance values for given motifs, and the inclusion of statistical tests when comparing motif enrichment. These changes have strengthened their conclusions. Only a few minor points remain:

-     The authors make a valiant effort to identify novel motifs through their use of STREME analysis, however the fact that known binding motifs (e.g. for Puf proteins) are not identified by their analysis is surprising – the authors should consider a more frank acknowledgement of this in the Discussion.
-     The histograms in supplemental figures are valuable additions, however the legends are nearly illegible.
-     It is not clear how bin size was chosen for these histograms. They seem unusually large considering enrichment score is a continuous variable.
-     The authors explain the challenges behind validating faithful 3′ cleavage in their library (reviewer #1, minor point #6), but the question still remains. References to previous studies that look at 3′ cleavage of mRNAs lacking the efficiency and positioning elements would help convince readers that the N50 sequences are unlikely to alter 3′ cleavage.

**Reviewer 2**

Thanks for this much improved revision.

**Authors’ response to reviewers**

- Reviewer #1: Only a few minor points remain:

- The authors make a valiant effort to identify novel motifs through their use of STREME analysis, however the fact that known binding motifs (e.g. for Puf proteins) are not identified by their analysis is surprising – the authors should consider a more frank acknowledgement of this in the Discussion.

**We added discussion in the Results section regarding the failure of our de novo motif discovery approach to identify the Puf protein binding sites (p. 17 of the revised version).**

- The histograms in supplemental figures are valuable additions, however the legends are nearly illegible.

**We remade the figure with substantially larger plots and legends for improved readability.**

- It is not clear how bin size was chosen for these histograms. They seem unusually large considering enrichment score is a continuous variable.

**There are tradeoffs between bin size and level of noise in the histogram due to low counts per bin. We binned at enrichment increments of 0.5 as this spacing spans the effective range of our data with a good number of bins -- approximately 30 -- while maintaining reasonable counts of variants per bin across sub-populations to avoid looking at noise. This is the smallest bin size we can afford for the lower-N cases of the (UA)5 motif and its mutants.**

- The authors explain the challenges behind validating faithful 3′ cleavage in their library (reviewer #1, minor point #6), but the question still remains. References to previous studies that look at 3′ cleavage of mRNAs lacking the efficiency and positioning elements would help convince readers that the N50 sequences are unlikely to alter 3′ cleavage.

**We expanded on this point in the Results describing our analyses of the positioning element and we cited relevant literature (p. 16 of the revised manuscript).**
